# Recent Advances on Polypyrrole Electroactuators

**DOI:** 10.3390/polym9090446

**Published:** 2017-09-14

**Authors:** Bingxi Yan, Yu Wu, Liang Guo

**Affiliations:** 1 Department of Electrical and Computer Engineering, The Ohio State University, Columbus, OH 43210, USA; yan.575@osu.edu (B.Y.); wu.2233@osu.edu (Y.W.); 2 Department of Neuroscience, The Ohio State University, Columbus, OH 43210, USA

**Keywords:** electroactivity, conducting polymer, counterion, delamination, high-speed

## Abstract

Featuring controllable electrochemomechanical deformation and excellent biocompatibility, polypyrrole electroactuators used as artificial muscles play a vital role in the design of biomimetic robots and biomedical devices. In the past decade, tremendous efforts have been devoted to their optimization on electroactivity, electrochemical stability, and actuation speed, thereby gradually filling the gaps between desired capabilities and practical performances. This review summarizes recent advances on polypyrrole electroactuators, with particular emphases on novel counterions and conformation-reinforcing skeletons. Progress and challenges are comparatively demonstrated and critically analyzed, to enlighten future developments of advanced electroactuators based on polypyrrole and other conducting polymers.

## 1. Introduction

Electroactive polymers have attracted ever increasing interests in driving biomedical devices [1,2,3,4], microactuators [5,6], and biomimetic robots [7,8,9,10]. Being one important class of electroactive polymers, conducting polymers (CPs) have been extensively studied in the past few decades with representative members including polypyrrole (PPy), polyaniline (PANI), poly (3,4-propylenedioxythiophene) (PProDOT), and poly (3,4-ethylenedioxythiophene) (PEDOT) [11,12,13,14,15]. CP electroactuators achieve mechanical deformation under electric fields via induced ion exchanges with their surrounding electrolyte and can operate at a relatively low voltage below 2 V in various ambient conditions [16]. In this class, PPy electroactuators stand out due to their capability of producing more significant strain and stress. Gaihre et al. compared the electroactivity of different CP electroactuators doped with the same counterion bis(trifluoromethanesulfonyl)imide (TFSI^‒^) and found that PPy/TFSI significantly outperformed other CPs like PEDOT/TFSI and PProDOT/TFSI [17]. The demands for versatile PPy electroactuators featuring high electroactivity and durability are intense in many fields including biomedical devices, flexible electronics and batteries [18,19,20,21].

In this past decade, tremendous efforts have been devoted to the optimization of PPy electroactuators for enhanced electrochemical stability, actuation speed, mechanical strength, and, most importantly, output strain and stress [15,20,21]. Strategies and approaches introduced in recent reports are enlightening to future developments of CP electroactuators. Therefore, we conduct this review on PPy electroactuators, emphasizing novel mechanistic designs leveraging counterion doping and conformation reinforcing. The selection of counterions and the design of structure have been the two foremost topics advancing PPy electroactuators. While counterion dopant accounts for electrochemomechanical deformation, the structure of an electroactuator determines its robustness against mechanical failures such as delamination, ripples, and cracks of multilayered CP electroactuators. With respect to these emerging techniques, this work will progress the discussion by considering: (1) how each PPy electroactuator works; (2) the pros and cons for each; and (3) whether the advantageous features could be combined with other emerging approaches for a better overall performance. In this way, recent advances are recapitulated to shed light on the further advancement of PPy electroactuators.

## 2. Special Doping Counterions for PPy Electroactuators

Anionic dopants as counterions are embedded into PPy films during electropolymerization to neutralize the positive charges arising as polarons and bipolarons in oxidized PPy chains [15]. Selection and optimization of these counterions have played an overriding role in pursuing high output strain and stress. Small dopants such as Cl^−^, F^−^, I^−^, Br^−^, ClO_4_^−^, BF_4_^−^, NO_3_^−^, SO_4_^2−^ and PF_6_^−^ are the first to be explored as counterions in CP electroactuators, achieving a typical output strain in the range of 1–3% [22,23,24,25]. Significantly enhanced output strain and stress have been attained by replacing these small counterions with larger ones. Given that large counterions are often immobile within the CP matrix during redox processes, cations in electrolyte are absorbed into an electroactuator, triggering a volumetric swelling during reduction, and then expelled during the subsequent oxidation, recovering the electroactuator. Equations (1) and (2) from Ref. [26] describe electroactuating redox reactions of PPy doped with small and large counterions, respectively; *s* refers to solid, *aq* refers aqueous solution, *A^−^* refers to anion, *MA^−^* stands for macroscopic anion, and *C^+^* refers to cation from electrolyte.

(1) $${\left(PPy^{0}\right)}_{s}+n{\left(A^{-}\right)}_{aq}+m\left(H_{2}O\right)\overset{red/oxide}{↔}{\left[{\left(PPy^{n+}\right)}_{s}{\left(A^{-}\right)}_{n}{\left(H_{2}O\right)}_{m}\right]}_{gel}+n{\left(e^{-}\right)}_{metal}$$

(2) $${\left[\left(PPy^{0}\right){\left(MA^{-}\right)}_{n}{\left(C^{+}\right)}_{n}\right]}_{s}\overset{red/oxide}{↔}{\left[\left(PPy^{n+}\right){\left(MA^{-}\right)}_{n}\right]}_{s}+n{\left(C^{+}\right)}_{aq}+n{\left(e^{-}\right)}_{metal}$$

**Figure 1:**
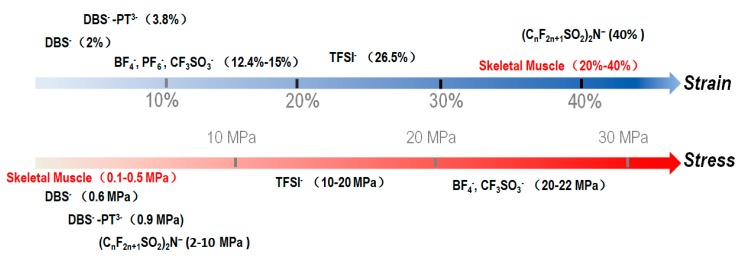
Output stain and stress of PPy doped with typical counterions.

Several theoretical models describe the electrochemical behaviors of PPy based on different assumptions. Otero et al. developed an electrochemically stimulated conformational relaxation (ESCR) model, attributing the electrochemical responses of a PPy film to its conformational change [27,28]. Posey and Morozumi proposed migration and diffusional models, treating PPy films as porous electrodes [29]. Feldberg introduced a capacitance model, explaining voltammetric results by means of capacitive currents [30]. Additional models could be found in Ref. [27]. Despite of these models, one widely accepted conclusion is that the strain and stress from a PPy electroactuator are primarily driven by ion exchanges between the PPy and electrolyte [26]. Figure 1 summarizes output strain and stress of well-studied PPy electroactuators doped with typical counterions.

Skeletal muscle is used as a reference in Figure 1. Myofibrils in skeletal muscles have rather large output strain but small stress, so they form small bundles known as fascicles that are further packaged and encapsulated by the perimysium [31,32]. This structure has inspired the development of electroactuators with a similar configuration for large output stress [33,34]. One observation derived from Figure 1 is a trade-off between strain and stress for the selection of a counterion. For example, (C_n_F_2n+1_SO_2_)_2_N-doped PPy features higher strain even comparing to skeletal muscles, but its output stress is very limited. On the other hand, BF_4_^−^- or CF_3_SO_3_^−^-doped PPy can generate rather large stress over 20 MPa, but can only produce a small strain. Table 1 further summarizes properties of PPy electroactuators doped with representative counterions to be discussed below.

### 2.1. Immobilized Large Anions as Dopants

Dramatically enhanced electroactivity has been reported when large counterions such as dodecyl benzene sulfonate (DBS^−^) are used as dopant to replace smaller ones such as ClO_4_^−^, CF_3_SO_3_^−^ or BF_4_^−^ in PPy electroactuators [17,53]. Volumetric and conformational changes of CP electroactuators are primarily induced and sustained by exchanges of hydrated ions together with osmotic flows [54,55]. Because hydrated cations, particularly Na^+^ (radius 3.6 Å), are much larger than hydrated anions (1.2 Å for hydrated Cl^−^) and can induce higher osmotic flows [56,57], cation-driven electroactuation is more mechanically significant than its anion-driven counterpart. To absorb cations into the polymer matrix, the CP backbones need to be electrically reduced. For CPs doped with small counterions that can freely exchange with the electrolyte solution under electric fields, however, the initial reduction causes the anionic counterions to flow out (leading to an initial shrinking of the material) before cations can be drawn from the electrolyte, which first counterbalances the initial shrinking and then causes swelling to the material. This phenomenon results in inconsistency in electroactuation and attenuation of the output strain and stress. Furthermore, unwanted exchanges of the doping counterions with other anions from the electrolyte disrupt chemical integrity and electrochemical stability of the CP [55]. In contrast, for CPs doped with large counterions that are immobilized in the polymer matrix, reduction can consistently draw hydrated cations from the electrolyte into the polymer matrix, resulting in a consistent and significant swelling [58,59], while a subsequent oxidation phase restores the polymer both mechanically and electrochemically [60,61]. A particular application of these CPs is in implantable biomedical devices for drug delivery or neuron recording, where the surrounding physiological fluids are enriched with cations such as Na^+^ [1,2,3,55].

One of the large counterions predominantly reported so far is DBS^−^. PPy/DBS electrochemically polymerized onto gold-coated tape has been widely employed as electroactuators operating in aqueous solutions [35,36], and this versatility has inspired consistent optimization of the PPy/DBS. Torop et al. synthesized carbide-derived-carbon (CDC)‒modified PPy/DBS and found the resulting film featured higher power efficiency and surface porosity, but its conductivity and electrochemomechanical deformation were compromised by CDC [62,63]. Recently, Zondaka et al. reported enhanced electroactivity and stability of PPy electroactuators via co-doping of DBS^−^ and multi-charged phosphotungstate anions (PT^3^^−^) [39]. The resulting film demonstrated 3.4 times higher strain and 5 times higher stress than a PPy/DBS film. High conductivity associated with fast ion exchanges was confirmed in the co-doped PPy and could be ascribed to (1) improved charging and discharging rates owing to immobilized highly charged PT^3^^−^ [40,41] and (2) the formation of ionic complexes that covalently bonded to the PPy backbone [42,43]. Comparing to prior PPy/TFSI electroactuators operating in an organic electrolyte solution, PPy/DBS offers a higher flexibility on the selection of electrolyte solutions. Phosphate buffered saline (PBS) or any Na^+^-enriched electrolyte can serve as the operating solution.

**Figure 2:**
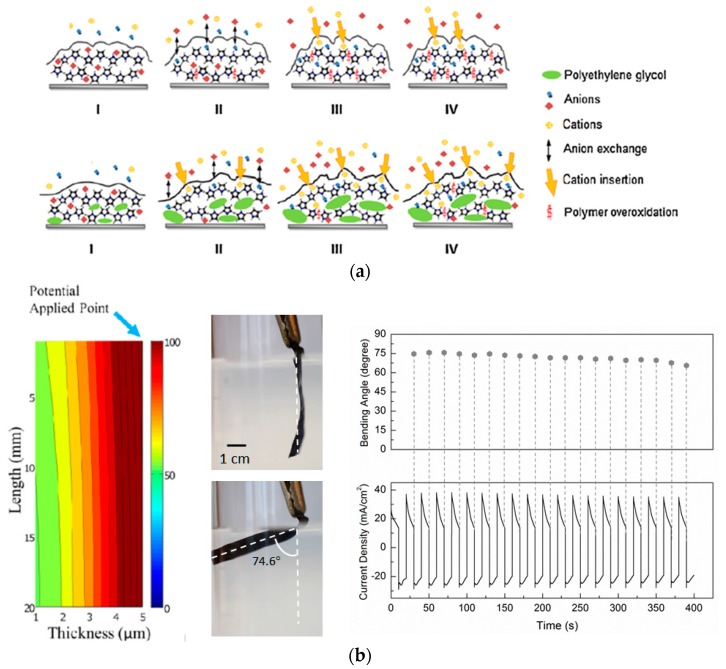
Electrochemomechanical deformation of polyethylene glycol (PEG)-modified PPy films. (**a**) Schematic illustration of the mechanism of PEG-retarded overoxidation of PPy, where I, II, III and IV refer to immersion time of 0.5 h, 2 h, 48 h, and 120 h in the testing solution, sequentially. Adapted with permission from Ref. [68]. Copyright (2011) Elsevier. (**b**) Simulation of voltage drop along the monolithic film (**left**), nonuniform bending of a PPy/PEG-borate strip in phosphate buffered saline (PBS) (**middle**), and degradation of strain and capacity when cycled by square waves of ±1 V amplitudes (**right**). Adapted with permission from Ref. [72]. Copyright (2017) IEEE.

Polyethylene glycol (PEG) as a neutral macromolecule has been incorporated into PPy electroactuators to improve their electrical and mechanical properties [64,65,66,67]. More importantly, incorporation of PEG proved effective to prevent overoxidation of PPy via restraining the electron release and rendering O_2_ inaccessible to PPy, thus leading to a better electrochemical stability against overoxidation [68]. Figure 2a illustrates the mechanism of suppressed overoxidation of the PEG-modified electroactuator, where PEG prevents rapid overoxidation by reducing the anion undoping process associated with polymer overoxidation. Another advantage of the PEG-modified electroactuator is fast strain response when PEG forms a complex with cations and accelerates the cation-driven swelling during reduction [69,70,71]. It should be pointed out that volumetric shrinkage under reduction is also slowed down by the same electron-refraining regime; hence such an electroactuator is particularly suitable for in vivo applications requiring high strain and durability rather than fast responses.

Inspired by PEG’s advantages and capability of forming an ionic complex with certain types of small ions, which as a whole can function as the doping counterion, we recently developed a series of PPy/polyol-borate composite films where long polyol chains or networks, such as PEG, polycaprolactone-block-polytetrahydrofuran-block-polycaprolactone (PCTC), or pentaerythritol ethoxylate (PEE), bridged by the borate groups were interwoven with the PPy chains during electropolymerization [20,73,74]. The PPy/PEE-borate film showed drastic actuation under a humidity gradient, while the PPy/PEG-borate and PPy/PCTC-borate were responsive to both humidity gradients and electrochemical stimuli. Electroactivity and water-responsive deformation of such composite films notably outperformed dual-responsive actuators reported earlier [34,75]. As an example, Figure 2b illustrates electrochemomechanical deformation of a PPy/PEG-borate film induced by cation exchanges. Upon immersion in PBS solution, water absorption initially induced a remarkable volumetric swelling and caused increased porosity in favor of the subsequent reduction-driven absorption of hydrated cations (mostly Na^+^), generating a pronounced bending strain. Considering their large and sustainable electroactivity, PPy/polyol-borate electroactuators are particularly suitable for biomedical applications where the only available electrolyte solutions are body fluids, such as driving an implantable insulin pump [51] and cardiac patches for treating myocardial infarction [31,32].

Another advantage of using immobilized macromolecular anions as dopant is the high biocompatibility of the resulting polymer composites, which proved comparable to bare gold and tissue culture polystyrene (TCP) when exposed to a variety of tissues and cells such as neurons [74,76,77], keratinocytes [78], epithelial cells [79], cardiac progenitor cells [80], skeletal muscle cells [81], rat pheochromocytoma cells [82], bone marrow-derived stem cells [83], blood [84], and so forth. These studies could be divided into two categories, those where PPy as an anticorrosion coating applied on metallic implants [85,86,87] and those where PPy films acting as a conducting substrate to deliver electrical stimuli to cells to promote their proliferation or differentiation rate [88,89,90]. Fahlgren et al. found PPy/DBS are more biocompatible than PPy doped with *p*-toluene sulfonated (pTS) or chondroitin sulfate (CS) to human primary osteoblasts [91], in good agreement with the observations reported by Stewart et al. on human neural stem cells [92]. The better biocompatibility was ascribed to the higher affinity between the cells and the smooth surface of PPy/DBS. Cells favoring small surface roughness were also observed by Fonner et al., who reported that polystyrene sulfonated (PSS) remarkably outperformed tosylate (ToS) and chloride as dopant, because PPy/PSS is smoother than PPy with the latter two dopants [93]. The underlying regime is that rough surfaces are overall hydrophobic and hence prevent strong attachment of cells. Apart from roughness and wettability, other factors such as surface charge and elasticity also affect biocompatibility of PPy substrates, and some previous reviews focusing on such issues could be found [94,95].

### 2.2. Freely Diffusible Counterions Combined with Organic Solvent Electrolytes

Similar to the large hydrated cations that can induce significant osmotic flows, some freely diffusible anionic dopants can also generate large deformation [26,47,96] with the help of organic solvents. A good example of such anion-driven electroactuators is PPy/TFSI. The film can produce significant output stain and stress when subjected to TFSI^‒^-enriched organic solvents such as propylene carbonate (PC), methanol or 2-propanol [48,49], making the electrochemomechanical performance of such electroactuators dependent on the solvent. Hara et al. reported that the volumetric strain decreased from 33.1% to 20.1% when the solvent was switched from PC to H_2_O and found that a H_2_O/PC ratio of 3/2 was the solution for the largest strain [50]. However, operation of such electroactuators requires existence of the large anions in the electrolyte, which cannot be met in biomedical applications, as these large anions are usually cytotoxic. Noteworthy, the solvent of electrolyte solution may alter the diffusion regime of counterions. For example, PPy/DBS has long been perceived as cation-driven electroactuators in aqueous solutions as discussed previously. Once immersed in an organic solvent such as PC or acetonitrile, however, the volumetric swelling due to solvent absorption eventually results in a more porous film and enables free diffusion of DBS^‒^ [37,38]. In this case, the PPy/DBS film turns into an anion-driven electroactuator.

### 2.3. Conformationally Transformable Anions as Dopants

**Figure 3:**
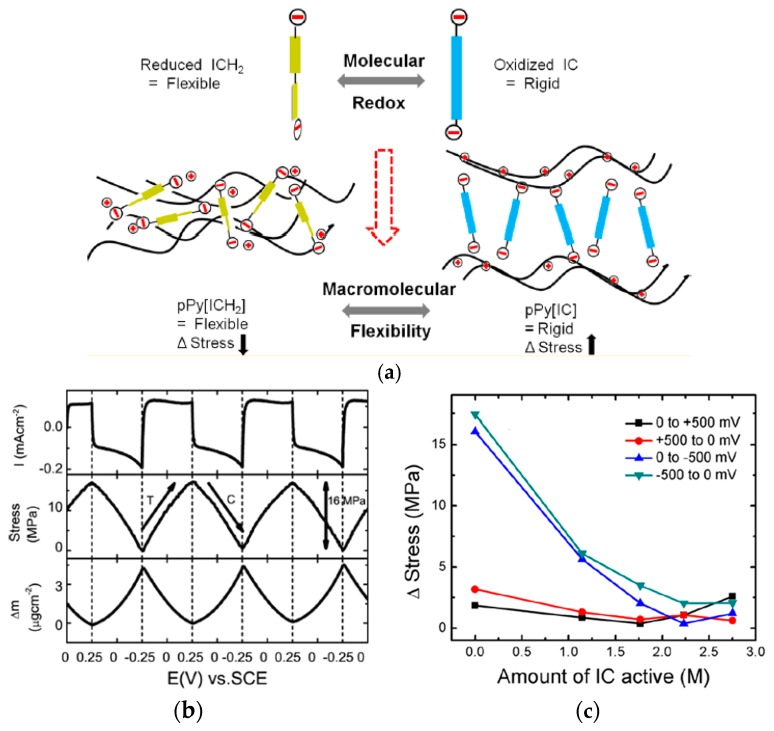
Actuation of PPy/IC electroactuators based on Au/Ti/Si substrates. (**a**) Schematic illustration of the redox-induced molecular conformation change of ICs in low-pH solvents. Adapted with permission from Ref. [44]. Copyright (2016) American Chemical Society. (**b**) Current density, stress and mass changes of PPy/IC during consecutive square-wave potential cycling (−0.25–0.25 V at 10 mV/s in 0.2 M LiCl solution). Adapted with permission from Ref. [45]. Copyright (2016) American Chemical Society. (**c**) Stress response as a function of active IC content in PPy; data collected from a PPy/IC film cycled from −0.5 V to 0.5 V. Adapted with permission from Ref. [44]. Copyright (2016) American Chemical Society.

Sen et al. recently reported a PPy electroactuator driven by the change of molecular conformation (Figure 3a), where dianionic indigo carmine (IC) molecules were incorporated into PPy as counterions during electropolymerization [44]. A common cation-driven behavior was observed from this composite film operating in a pH neutral electrolyte solution (0.2 M LiCl), with a maximum stress of 16 MPa (Figure 3b). However, when hydrochloric acid (0.2 M HCl) was gradually added into the electrolyte solution, bulky IC molecules featuring an inherent redox activity became electrochemically responsive, meaning the counterion itself turned rigid (oxidized) or flexible (reduced) following a regime as shown in Figure 3a [45,46]. This altered molecular structure was controlled by an external voltage in low pH solution, eventually giving rise to a remarkable macroscale stress response during redox process—a stress of 16.2 MPa was recorded from an electroactuator cycled at ±0.25 V (Figure 3b).

As seen in Figure 3b, the redox-regulated molecular stiffness brought about a notable stress response from the electroactuator. Specifically, given that reduced IC molecules were flexible in low pH electrolyte solutions, PPy chains became more intensively packed during reduction thus causing macroscale shrinkage which is contradictory to the predominant cation-driven swelling. Likewise, oxidation-induced volumetric shrinking of the cation-driven film may also be compromised by the expanding oxidized IC molecules. In either case, switching molecular stiffness diminished the overall cation-driven deformation and stress response in the low pH solution, an unwanted effect which could be even more pronounced for high contents of IC dopants as seen in Figure 3c. Despite the compromised stress response, the intriguing mechanism of electrochemomechanical change in the PPy/IC film offers the possibility of a new type of pH sensors based on subtle stress changes. Additionally, when operating in neutral LiCl or NaCl solution, the inactive ICs acting similarly to typical immobilized large counterions were able to induce a large stress response, which makes ICs suitable for electroactuators that need to perform rapid and strong force feedback in a limited space.

## 3. Delamination in Layered PPy Electroactuators

Designs of PPy electroactuators encompass a monolithic, bilayered, or trilayered structure [22,97,98]. Monolithic and bilayered implementations are primarily used in applications involving a supporting liquid electrolyte (either aqueous or organic), while trilayered ones are employed with an ionic gel electrolyte sandwiched between two PPy films for operation in air [23,99,100,101]. Due to longitudinal voltage attenuation along a semiconducting CP strip, a monolithic electroactuator’s tail usually generates smaller strain than its upper section [72], resulting in a notable strain gradient along the strip (Figure 2b). Another limitation of monolithic CP electroactuators is the time hysteresis on strain generation due to inherent mechanical resistance, which prevents their use in high-speed applications. To address these issues, in bi- and trilayered CP implementations, a flexible and much more conductive film, such as a strip of gold-coated Kapton^®^ tape or polyvinylidene fluoride (PVDF) membrane, has been employed as a substrate to reinforce the mechanical strength and deliver a uniform current density to the CP film [102,103,104,105]. This approach is effective to achieve quick and uniform bending strain, but brings about the risk of delamination which has become a major challenge for the devolvement of multilayered electroactuators. Therefore, strategies to ameliorate or eliminate delamination-associated mechanical failures have been a key topic in the design and assembly of layered CP electroactuators. This section delineates innovative strategies targeting these long-existing challenges.

### 3.1. Mechanism and Models for Cracking and Delamination

**Figure 4:**
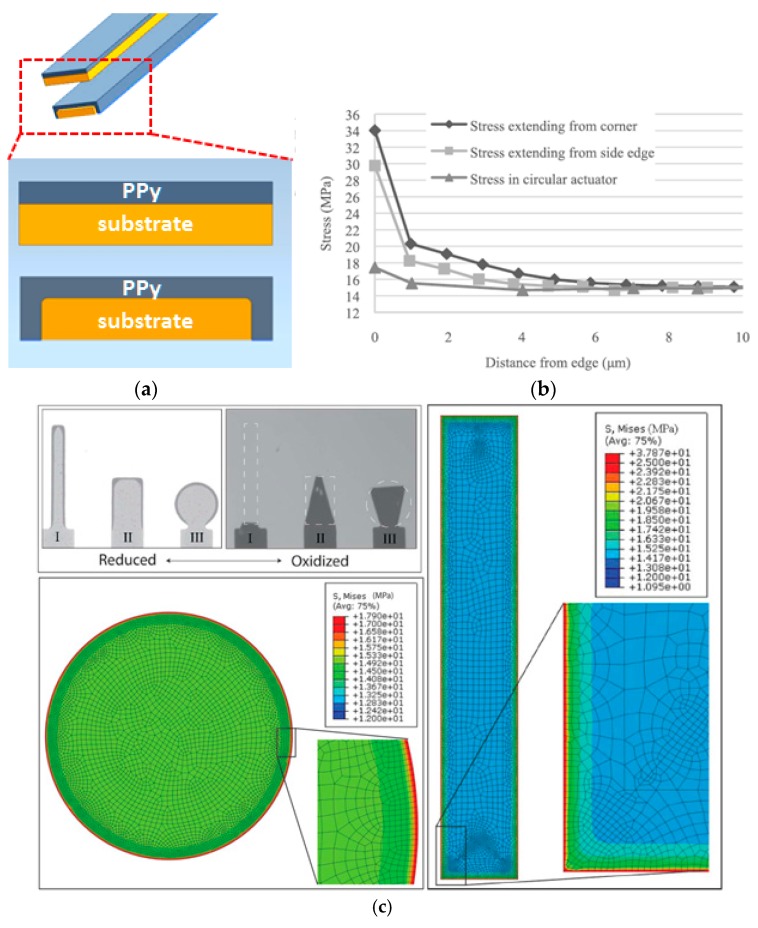
Delamination of bilayered PPy electroactuators. (**a**) U-shaped edge enclosures preventing delamination from a strip substrate; (**b**) Interfacial stress as a function of distance to edges. Adapted with permission from Ref. [108]. Copyright (2015) IEEE. (**c**) Simulation and test on delamination for substrates of various geometries. The color bar from blue to red indicates increasing interfacial stress. Adapted with permission from Ref. [108]. Copyright (2015) IEEE.

Film delamination could occur as a result of excess interfacial stress arising from thermal expansion mismatch [106], growth process [107], or strain mismatch [108] during redox reactions, with strain mismatch being dominant in many PPy-gold bilayers. In bilayered electroactuators, a gold or platinum layer (20‒50 nm) fabricated by magnetic sputtering or electron beam evaporation has been preferentially used as a metal coating for the subsequent electropolymerization of CP films. Small polymerization current, rough electrode surface, and low reaction temperature are common considerations to achieve mechanically robust bilayers [109,110,111,112,113,114]. However, we observed that PPy films electropolymerized on a metal-coated surface usually extended onto the uncoated edges of the thin substrate, forming a U-shaped enclosure at the edge as illustrated in Figure 4a. This unique structure, though not much discussed, might play a pivotal role against delamination. Accordingly, when the order of the fabrication processes is inversed by coating a metal film onto a pre-synthesized PPy film, the structure is prone to delamination that initiates from the unenclosed edges. Recalling that most CP films reported so far are deposited on pre-tailored metal-coated strips, the risk of delamination might have been mostly suppressed by the U-shaped enclosure even in the absence of a strong adhesion between the CP film and substrate.

Ho et al. investigated the delamination mechanism of PPy-gold bilayers using finite element analysis [108]. In their study, PPy/DBS films of 1.5 µm thickness contracted volumetrically during oxidization by expelling cations, initializing a self-liftoff process. In Figure 4c, the narrow rectangular bilayer detached faster than the wider rectangular and the circular bilayers under the same operating conditions. Simulation results indicated that the maximum magnitude of redox-induced interfacial stress was 15 MPa at the center, but increased to 34 MPa and 18 MPa at the edge of the rectangular and circular bilayers, respectively. Therefore, for a given area, bilayers with a larger perimeter tended to liftoff faster than those with smaller ones. These results suggest that a bilayered strip electroactuator—even remains one of the most widely studied structures so far—is a mechanically vulnerable configuration against delamination [106,111,112]. Therefore, the need for conductive skeletons featuring stronger interfacial adhesion is urgent.

### 3.2. Substrates for Delamination-Free Electroactuators

Conductive skeletons including nanoporous gold, multi-walled carbon nanotubes (MWCNTs), and interpenetrating polymer network (IPN) have been exploited as reinforcing frameworks in CP electroactuators [115,116,117]. Unlike the aforementioned counterions that are doped in PPy for large output strain and stress, conductive frameworks are used to improve the actuation speed that otherwise is restricted by the relatively slow transport of ions in the polymer. This strategy arises naturally since conductive skeletons of submicron porosity can help transmit the actuating electric currents directly to PPy while simultaneously providing large interfacial areas for accelerated ion exchanges between the actuator and electrolyte solution. Thus, a high conductivity could still be preserved in the composite film even when PPy is reduced to neutral. However, the output strain of such skeleton-reinforced electroactuators is generally compromised due to the added material resistance.

**Figure 5:**
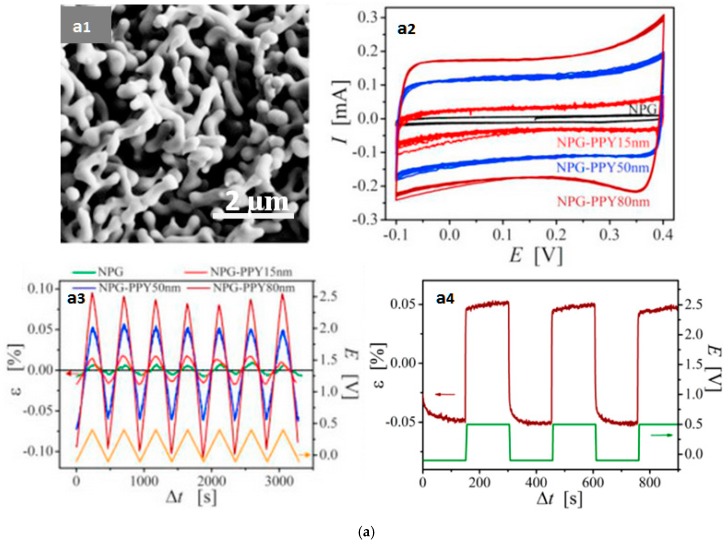

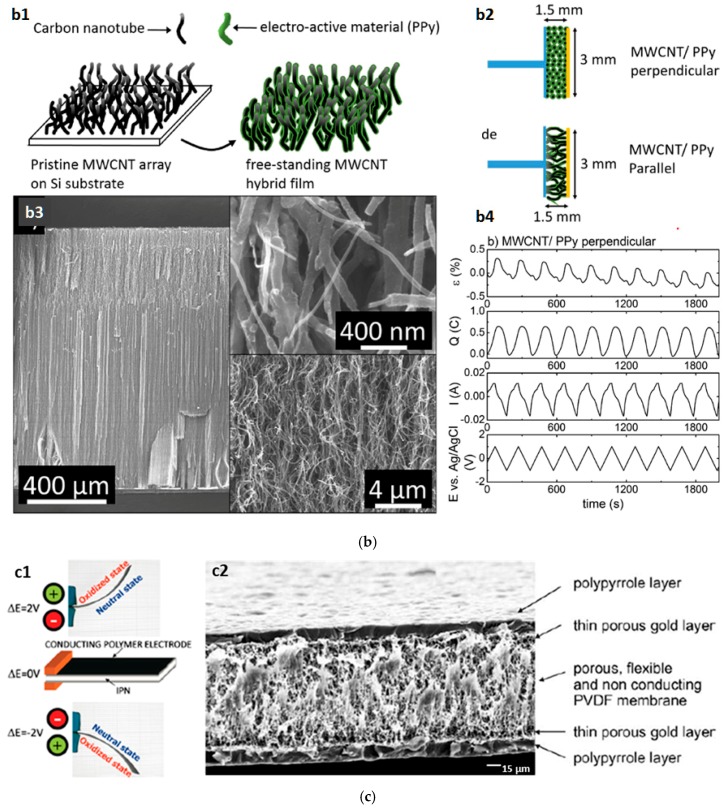
PPy electroactuators involving conductive skeletons. (**a**) Scanning electron microscope (SEM) morphology of nanoporous gold with PPy coating (**a1**), cyclic voltammograms (**a2**), strain response during triangular potential stimulation (**a3**), and square-wave potential stimuli (green curve in (**a4**)) cycled from −0.1 V to 0.4 V in 1M HClO_4_. Adapted with permission from Ref. [117]. Copyright (2017) Elsevier. (**b**) Schematic of the synthesis procedure (**b1**), two types of orientation for multi-walled carbon nanotubes (MWCNTs) (**b2**), cross-sectional SEM of a PPy-MWCNTs composite film (**b3**), and electroactive response of the composite electroactuator in a potential cycle of −1 V to 1 V, tested in 0.5 M KCl solution (**b4**). Adapted with permission from Ref. [125]. Copyright (2015) John Wiley and Sons. (**c**) Operation of an interpenetrating polymer network (IPN)-based PPy electroactuator (**c1**), adapted with permission from Ref. [126]. Copyright (2011) AIP Publishing LLC, and a cross-sectional view of the layered structure (**c2**), adapted with permission from Ref. [127]. Copyright (2006) Elsevier.

Nanoporous metal skeletons are bulky sponges distinguished by their capability to convert chemical and electrochemical energy directly into mechanical responses via absorbate- or charge-induced changes in surface stress [118,119,120]. Direct electrodeposition of PPy on nanoporous gold (NPG) is an effective approach to combine the high mechanical strength of NPG and the large tensile tolerance of conjugated polymers. Wang et al. reported a composite film comprising NPG encapsulated with a conformal coating of PPy (Figure 5a) and confirmed a linearly proportional correlation between PPy thickness and the pseudocapacitance of the film [117]. When cycled from −0.1 V to 0.4 V, the 80 nm PPy coating demonstrated a larger amount of charge stored in the composite than thinner coatings (Figure 5(a2)). By measuring the length variation of a strip of the composite material, they further confirmed that the electrochemomechanical deformation increased as PPy grew thicker (Figure 5(a3)). Specifically, skeletons coated with 80 nm PPy demonstrated a volumetric strain of 0.19%—12 times larger than that of pristine, uncoated skeleton (0.015%). The feature time delay (*t_½_*) of such reinforced composite electroactuators could be as small as 1 s, meaning that it could output 50% of its maximum strain almost instantly as the redox process began.

Apart from bulky skeletons, incorporation of nanoscale CDC particles or MWCNTs is another approach to enhance the conductivity, mechanical strength, and actuation speed of PPy electroactuators [121,122,123,124]. A PPy/DBS-CDC hybrid electroactuator could output 13% diametral strain under reduction. Although this was smaller than that of a PPy/DBS electroactuator (25%), the CDC-modified electroactuator presented high actuation efficiency that was characterized by assessing strain induced per charge—1.17% per mC was recorded for PPy/DBS-CDC, outperforming 0.6% per mC for PPy/DBS.

Schnoor et al. synthesized a composite film consisting of oriented MWCNTs (38 nm in diameter, 1500 μm in length) that were coated and stabilized by 30 nm PPy (Figure 5(b1)) [125]. In Figure 5(b3), it can be clearly seen that each individual nanotube was coated by PPy. A strain plateau showed up from a MWCNT-PPy film in Figure 5(b4) when the applied potential reached −1 V, but was not observed from pristine PPy films, meaning MWCNT-modified PPy reached strain saturation faster. Comparing to the PPy with MWCNTs oriented in parallel, the hybrid film demonstrated ten times larger strain when the MWCNTs were oriented perpendicular to the loading direction (Figure 5(b2)). In this way, an anisotropic strain response was achieved by modulating the aligning direction of MWCNTs in this film.

IPN (Figure 5(c1)) is another type of skeleton that has been used in delamination-free electroactuators [126,127]. It is distinguished by three major advantages comparing to NPG and MWCNTs: (1) IPN has smaller inherent stiffness and thus allows large bending strain; (2) its capability to accommodate ionic gel replenished with electrolyte solution enables controllable electroactuation in air; and (3) IPN can be fabricated and patterned via conventional microfabrication techniques such as spin-coating, photolithography, and ions beam etching; therefore, it is particularly attractive for use in microsystems.

**Figure 6:**
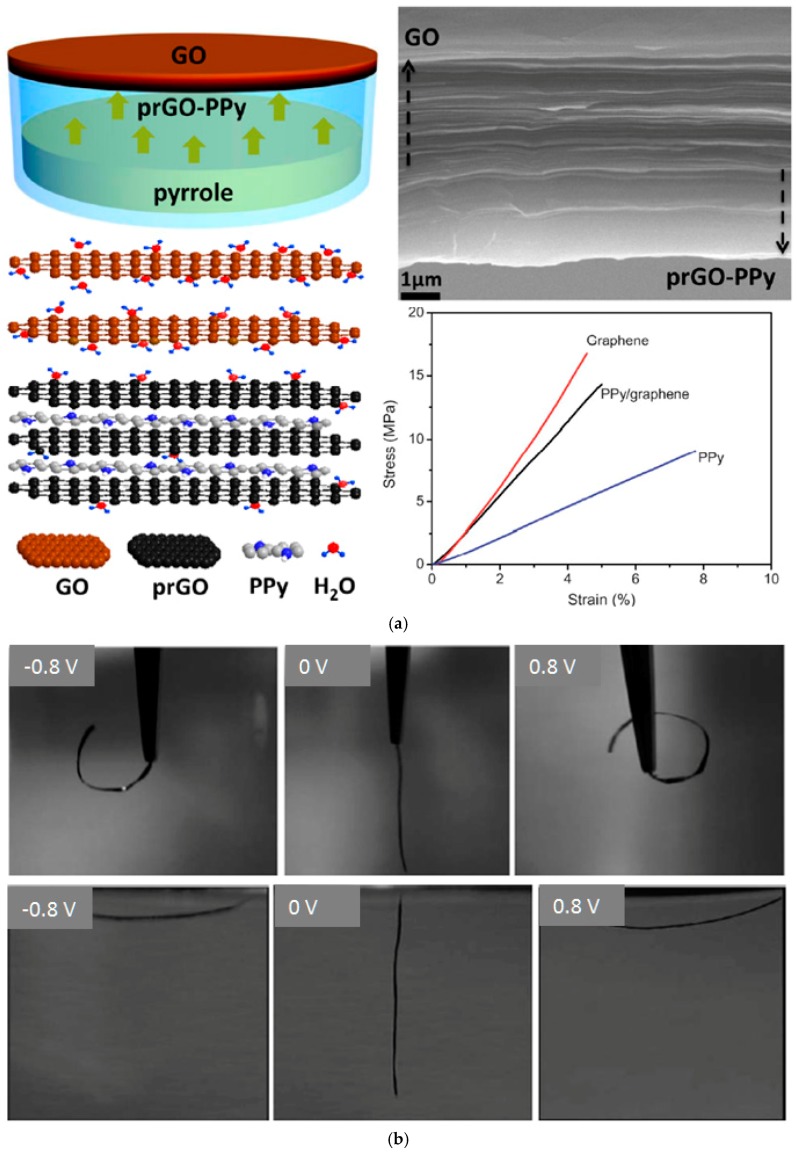
PPy/graphene oxide (GO) electroactuators. (**a**) Fabrication process (**left top**), cross-sectional view (**right top**), schematic illustration of dual-responses to water and electrochemical stimuli (**left bottom**), and tensile test showing the strain-stress curve of the composite film (**right bottom**). Adapted with permission from Ref. [134]. Copyright (2016) American Chemical Society. (**b**) Bending test of a PPy/GO strip electroactuator with the holding tip immersed (**upper**) or above (**lower**) the electrolyte solution. Adapted with permission from Ref. [132]. Copyright (2012) The Royal Society of Chemistry.

Multilayered graphene serving as soft conductive substrates has been developed to achieve high and fast strain responses [128,129,130,131]. To enhance the interfacial bonding of PPy and graphene layers, O_2_ plasma treatment was applied to produce hydrophilic surfaces, which also facilitated the subsequent electropolymerization process [132]. Another effective route is to insert a sulfonated graphene adhesion layer between the PPy layer and the graphene substrate [133]. Jiang et al. reported a facile one-step fabrication of PPy on partially reduced graphene oxide (prGO). The resulting film featured notable strain responses to both humidity gradients and electric stimuli [134]. As shown in Figure 6a, the graphene oxide (GO) film was exposed to pyrrole monomer vapor to form the prGO-PPy film at room temperature, where pyrrole vapor readily attached and thereafter was oxidized by GO, while the polymerization of pyrrole partially reduced GO into prGO at the polymer-graphene interface. A review on this method known as Vapor Phase Polymerization (VPP) can be found elsewhere [135]. Because oxygen-related functional groups existing in the stacked GO films absorb water molecules more efficiently than the PPy side, this actuator demonstrated water-responsive deformation when subjected to a humidity gradient. In addition, the rather high conductivity (over 1000 S/cm) arising from conductive graphene layers ensured a fast and uniform bending, whereas, in contrast, the monolithic PPy strip only swayed under redox cycles with limited strain observed from its tail (Figure 2b). The electroactuator demonstrated a reversible bending angle around 40° when cycled in 1M NaClO_4_ solution between −0.8 V and 0.8 V. Comparing to PPy/polyol-borate, electrochemomechanical deformation of this electroactuator was faster yet smaller due to extra mechanical resistance of the micrometer-thick GO substrate. Such PPy/graphene bilayers showed a higher Young’s modulus than pristine PPy, and, therefore, a larger tensile stress was recorded at the same strain as seen in Figure 6a.

**Figure 7:**
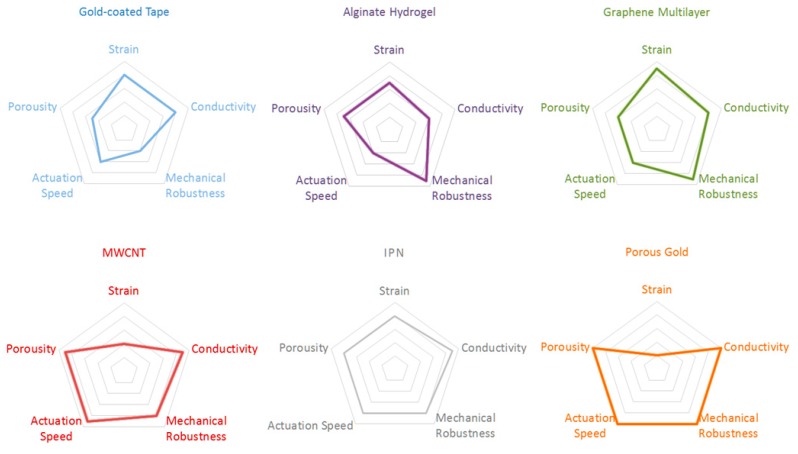
Radar diagrams of various substrates featuring different combinations of properties.

A schematic summary of substrates with different combinations of properties is shown in Figure 7. Considering the varying experimental conditions in different studies, such plots only manifest pros and cons of each substrate qualitatively rather than quantitatively and are intuitively helpful to guide readers to identify the appropriate substrate type for a combination of desired properties. In each plot consisting of five concentric pentagons, from the outmost to the innermost, properties vary from excellent to poor. Here, mechanical robustness is evaluated primarily based on vulnerability to failures such as delamination, cracking, and the maximum stretching tolerance. Actuation speed depends on the overall rate of ion exchanges, which is correlated to porosity and substrate conductivity. Strain represents either bending strain or volumetric expansion/contraction ratio.

Porous gold and aligned MWCNTs can serve as conductive skeletons for high-speed electroactuators working at small strain. The cross-linked metal ligaments effectively suppressed mechanical failures including cracking, delamination and creeping effect [136,137]. Furthermore, the highly porous configuration is particularly desired for emerging PPy-based photocatalysts and supercapacitors that require large specific areas [138,139,140,141,142]. Flexible GO has smaller stiffness comparing to other skeletons and substrates, and thus electroactuators based on graphene layers present larger bending strain. Likewise, IPN-based electroactuators generate large strain at high actuation speeds, while also offering the capability to accommodate ionic gels in place of electrolyte solutions. This property makes it a promising candidate for multilayered electroactuators operating in air, though processability of IPN as substrates remains inefficient to date. In PPy-alginate, PPy was electrodeposited through the porous hydrogel and then peeled off the substrate together with the hydrogel base, where an interpenetrating region was formed [52,143]. Water-saturated alginate hydrogel is soft, but turns stiff once dried out. This alterable stiffness enabled the electroactuator to output moderate strain while preserving sufficient mechanical strength against tensile and shearing forces. Last, the costs of these novel substrates, though not evaluated in this study, are generally higher than that of the gold-coated tapes, and thus the pursuit of cost-effective substrates of comparable properties is likely to be another continuing topic in this field.

## 4. Conclusions

This paper reviewed recently reported PPy electroactuators involving novel counterions and substrates. Intriguing features were introduced, compared, and critically analyzed. Below we provide some suggestions:

1. For monolithic PPy electroactuators designed to work in aqueous solutions, particularly bio-relevant solutions such as PBS, PPy films doped with large counterions especially polyol-borate could provide outstanding strain and electrochemical stability and are thus promising for various biomedical devices. Moreover, alginate hydrogel could serve as an ideal substrate in such electroactuators to further improve its processability and reduce the risks of being broken and torn during fabrication.
2. In solutions where supportive anions like BF_4_^−^, PF_6_^−^, CF_3_SO_3_^−^ or TFSI^−^ are accessible, a combination of a graphene or IPN substrate with counterions like DBS^−^‒PT^3−^, CF_3_SO_3_^−^ or TFSI^−^ may result in delamination-free electroactuators of superb electroactivity. This could also be the first choice for multilayered electroactuators involving ionic gels replenished with required anions.
3. For applications requiring fast electroactuation and large output stress, PPy incorporated with ICs or aligned MWCNTs could be a good candidate, though the output strain of such electroactuators is generally limited. It is worth noting that the extremely large porosity and surface area arising from these dopants are highly favored by supercapacitor and photocatalytic applications.

In summary, nanoporous skeletons, ICs and aligned MWCNTs can effectively improve the electroactuation speed and output stress with limited deformation. Monolithic PPy/polyol-borate electroactuators demonstrate large output strain along with enhanced electrochemical and electromechanical stabilities. Flexible graphene and IPN layers are ideal substrates for PPy electroactuators of remarkable output strain and stress. Given that electroactuators developed so far remain refrained by a number of limitations, a good strategy is to combine complementary counterion and substrate together to achieve the desired properties by specific applications.

## Figures and Tables

**Table 1:** Electrochemomechanical properties of PPy electroactuators doped with representative counterions.

	DBS	DBS-PT	IC	TFSI	Polyol-Borate
**Testing Solution**	NaDBS, aq.	LiTFSI, aq.	LiCl, aq.	LiTFSI, org.	Saline, aq.
**Strain**	2% (*L*), 10.4%(*B*)	3.8% (*L*)		22.9‒34% (*V*)	118% (*B*)
**Stress**	0.62 MPa	0.86 MPa	16 MPa	6.7‒10.5 MPa	
**Actuation Speed**	0.05 Hz	0.05 Hz	0.02 Hz	0.01 Hz	0.04 Hz
**Efficiency**	0.6%/mC	1.9%/mC			
**Conductivity**	0.5 S/cm	8.5 S/cm		129 S/cm	92.2 S/cm
**Tensile Tolerance**	15 MPa			27 MPa	13.5 MPa
**Reference**	[35,36,37,38]	[39,40,41,42,43]	[44,45,46]	[33,47,48,49,50]	[51,52]

PT refers to phosphotungstate anions, aq. indicates aqueous solution, and org. means organic solvents. *L*, *V*, and *B* represent linear, volumetric, and bending strain, respectively. All actuation speeds are given based on the highest frequency reported, at which the maximum reversible strain is achieved.
